# SARS-CoV-2 Infection Prevalence in the Population of South-Eastern Poland

**DOI:** 10.3390/diagnostics11112115

**Published:** 2021-11-15

**Authors:** Ewa Stępień, Marcin Koleśnik, Katarzyna Mitura, Maria Malm, Bartłomiej Drop, Marian Jędrych, Małgorzata Polz-Dacewicz

**Affiliations:** 1 Department of Virology with SARS Laboratory, Medical University of Lublin, 20-093 Lublin, Poland; ewa.stepien@umlub.pl (E.S.); katarzyna.mitura@umlub.pl (K.M.); malgorzata.polz-dacewicz@umlub.pl (M.P.-D.); 2 Department of Medical Informatics and Statistics with E-learning, Medical University of Lublin, 20-090 Lublin, Poland; maria.malm@umlub.pl (M.M.); bartlomiej.drop@umlub.pl (B.D.); marian.jedrych@umlub.pl (M.J.)

**Keywords:** SARS-CoV-2, COVID-19, Poland, cancer patients

## Abstract

COVID-19 outbreak began in Wuhan, China, and has spread to other continents, including Europe, placing pressure on healthcare systems. Poland is one of the European countries with the highest number of SARS-CoV-2 infections and COVID-19-related deaths. The aim of this study was to analyze the presence of SARS-CoV-2 in the population of south-eastern Poland. The correlation between viral infection and demographic data (gender, age, place of residence) and cancer was also investigated. A total of 44,801 samples were tested, of which 4862 cases were diagnosed with SARS-CoV-2 infections. A total of 14,970 samples were tested in cancer patients. The RT-PCR method was used to detect viral nucleic acid. In this study, significantly, the highest rate of virus detection was among people living in Lublin and the lowest among people living in a small town (*p* < 0.0001). Moreover, there was no significant relationship between sex and the frequency of virus detection. The highest number of SARS-CoV-2 infections was observed in the age groups 10–19, 20–29, 30–39, and 90+ (*p* = 0.0001). In cancer patients, the percentage of positive cases was significantly lower than in the rest (*p* = 0.0001).

## 1. Introduction

Coronaviruses are RNA viruses that infect both humans and animals. In December 2019, SARS-CoV-2 (severe acute respiratory syndrome coronavirus 2) was identified as the cause of an acute respiratory disease termed COVID-19 (coronavirus disease 2019). The virus is transmitted by airborne droplets. The high contagiousness of SARS-CoV-2 and the high frequency of international travel caused COVID-19 to spread worldwide, and on 11 March 2020, the World Health Organization (WHO) declared a pandemic. Signs of infection appear after an incubation period of 1 to 14 days (usually around 5 days). The most common symptoms of the disease are fever, cough, shortness of breath, and breathing problems. The disease may also be accompanied by loss of smell and taste, muscle aches, and fatigue. In many cases, SARS-CoV-2 infection is asymptomatic, which significantly affects the spread of this virus in public spaces. SARS-CoV-2 poses a particular threat to immunocompromised people, for example, cancer patients who are treated with various methods, such as chemotherapy or radiotherapy. Despite ongoing vaccination campaigns around the world, coronavirus outbreaks are on the rise in large part due to the emergence of new variants of SARS-CoV-2 [1,2]. 

According to WHO data, 229,373,963 confirmed cases of COVID-19 were reported globally, including 4,705,111 deaths. The European Union countries with the highest number of cases of SARS-CoV-2 infection and associated deaths include France, Spain, Italy, Germany, and Poland [3,4]. The current population of Poland is 38 million and is one of the largest in the European Union. So far, almost 3 million infections and over 75,000 deaths related to COVID-19 have been reported in Poland [3]. The first case of COVID-19 in Poland was confirmed on 4 March 2020. Since then, there have been 3 waves of the pandemic in Poland: March–June 2020, October–December 2020, and March–May 2021. During the COVID-19 pandemic, special hospitals for patients suffering from COVID-19 were established in Poland, and general-profile hospitals were transformed into COVID hospitals. Each patient with COVID-19 requiring hospitalization had the option of free hospitalization under the public health care system. Due to differences in health care systems and the number of tests for SARS-CoV-2 infections in individual countries, data on the trend of COVID-19 infections due to socio-demographic features and the presence of diseases such as cancer are necessary [5].

The aim of the current research was to estimate the number of SARS-CoV-2 infections in south-eastern Poland. This study is retrospective.

## 2. Materials and Methods

### 2.1. Sample Collection

Nasopharyngeal, throat and nose, or nose swabs were collected in April 2020 to 30 March 2021. Samples were taken from both symptomatic and asymptomatic patients at drive-thru points and hospital emergency rooms in the Lublin area. Oncological patients were admitted as persons hospitalized in the Lublin Voivodeship. Demographic data including age, gender, and place of residence of participants were collected. A total of 44,801 samples were tested, of which 59.1% were females and 40.9% males. Due to the place of residence, 20,493 (45.74%) people were from Lublin area, 9149 (20.42%) from a small town, and 14,929 (33.32%) from the rural areas. The number of cancer patients in the study group was 14,970 (33.41%).

### 2.2. Laboratory Analysis

Viral RNA isolation was performed by automated nucleic acid extraction using the Nucleic Acid Extraction Kit (TANBead, Taiwan Advanced Nanotech Inc., Taoyuan, Taiwan) according to the manufacturer’s instructions. RNA was extracted from 300 µL of material.

Novel Coronavirus (2019-nCoV) Real-Time Multiplex Kit (3-gene detection) (Liferiver, Shanghai, China) was used to qualitatively detect SARS-CoV-2 RNA in upper respiratory samples by real-time PCR using CFX96 (Bio-Rad, Hercules, CA, USA). This kit detects 3 genes: ORF1ab, N gene and E gene, and internal control (IC). The conditions consisted of 1 cycle of 10 min at 45 °C, 1 cycle of 3 min at 95 °C, and followed by 45 cycles of 15 s at 95 °C and 30 s at 58 °C (fluorescence was measured at 58 °C). The analytical sensitivity of this assay was 1 × 10^3^ copies/mL. The result was analyzed using CFX96 (Bio-Rad). Ct (cycle threshold) is the number of cycles required for a fluorescent signal to pass the threshold (exceeds background level). Ct levels are inversely proportional to the amount of target nucleic acid in the sample (the lower the Ct level, the higher the amount of target nucleic acid in the sample). A result was interpreted as positive if the Ct value represented a positive detection signal, defined as Ct ≤ 43 for at least 2 of the 3 detected genes. Internal control detection was not required when at least two of the three genes detected were positive. In case of a negative result for all three genes and a positive detection signal for the internal control, defined as Ct ≤ 35, the result was interpreted as negative. 

### 2.3. Statistical Analysis

Statistical analysis was performed to investigate the relationship between SARS-CoV-2 infection and the clinical and demographic characteristics of patients. Pearson’s chi-square test [6] was used in the study. 

### 2.4. Ethics

This study was approved by the Bioethics Committee at Medical University of Lublin, Poland (Resolution No. KE-0254/104/2020 from 27 May 2021).

## 3. Results

### 3.1. Socio-Demographic Characteristics of the Studied Population

Details of the socio-demographic characteristics of the studied population are presented in Table 1.

### 3.2. The Presence of SARS-CoV-2 Due to Sociodemographic Features and Cancer

Table 2 presents the socio-demographic characteristics of the studied population and the percentage of positive and negative cases of SARS-CoV-2 infection taking into account the division into cancer and non-cancer patients.

### 3.3. Characteristics of SARS-CoV-2 Infection by Age and Place of Residence

Using RT-PCR to detect the virus in samples, SARS-CoV2 RNA was detected in 4862 (10.85%) cases. The highest percentage of infections was recorded in the age range of 10–19 years: 25.8% in Lublin; 13.3% in rural areas, and 4.8% in a small town. In a small town, 11.3% were infected with SARS-CoV-2 in the age of 20–29. The lowest percentage of people infected were in the age group 70–79: 4.5% in rural areas, 5.8% in a small town, and 7.8% in Lublin, respectively. In the population over 80 years of age, there has been an increase in infections. The infection rate in the age group 90+ was, respectively 13.5% in Lublin, 17.6% in a small city, and 20% in the rural areas.

### 3.4. Prevalence of SARS-CoV-2 Infection in South-Eastern Poland between April 2020 and March 2021 by Place of Residence

During the period April 2020–March 2021, from April 2020 to August 2020, several percentages of cases were recorded. There was a noticeable rise since September 2020. In October 2020, the infection rate was 24% for Lublin, 19.1% for the countryside, and 14.5% for a small town. In November 2020, the number of infections was 26.2% in Lublin, 20.1% in rural areas, and 14.1% in small towns. In December 2020, a drop was observed, even higher in January 2021: 13.4% Lublin, 95% village, and 4.5% small town, respectively. In February 2021, the infection rate was even lower: 10% in Lublin and about 4% in rural areas and small towns. In March 2021, infections increased again by 18.5% in Lublin, 7.8% in the countryside, and 6.3% in a small town, respectively.

### 3.5. Prevalence of SARS-CoV-2 Infection by Age and Sex

In people over 70 years of age, infection was detected more often in females (11.6%) than in males (9.3%). At age 0–9, the virus was detected more often in females (13%) than in males (9.3%).

### 3.6. Prevalence of SARS-CoV-2 Infection in Oncology Patients between April 2020 and March 2021

Oncological patients are an important group. In these patients, the first positive results were obtained at the turn of August 2020 and September 2020. In October 2020, infections peaked in 11.8% of females and 10.2% of males. Then the percentage dropped, and in February 2021 and March 2021, the percentage of those infected was around 2%.

### 3.7. Characteristicsof SARS-CoV-2 Infection in Oncology Patients by Age and Sex

In the group of cancer patients under 20 years of age, only positive results were found in females (3.6%); in the age of 20–29, the percentage of SARS-CoV-2 infection in females and males was similar and amounted to 7.5%. At the age of 60–69, a higher number of infections was recorded in males, and in the age group 80+, females (3.7%) prevailed over males (2.5%).

## 4. Discussion

For several months after the first case of COVID-19 was reported in Poland, the number of infections in this country was among the lowest in Europe. The reason for this was the fact that in Poland compared to other European countries, restrictions were introduced early to limit the spread of infection in public spaces. An example of such actions was the ban on mass events, restriction of catering and sports facilities, the introduction of controls on the Polish borders, the declaration of an epidemic, and the obligation to quarantine people returning from abroad. The abolition of sanitary rules from 4 May 2020 contributed to the subsequent increases in infections [7,8]. 

**Figure 1:**
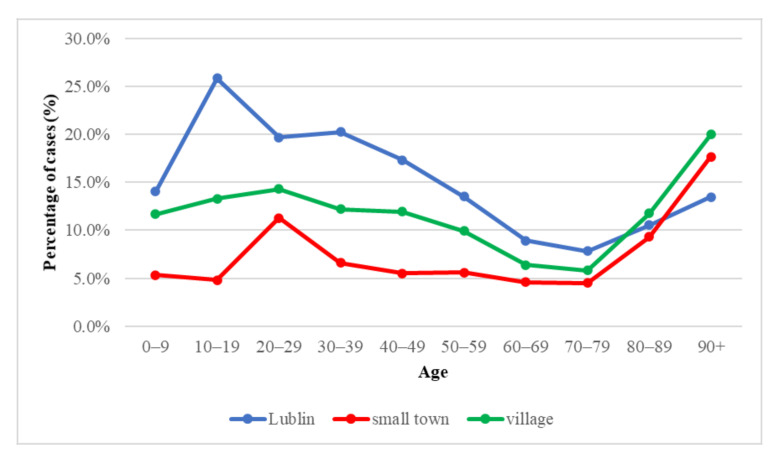
SARS CoV-2 infection by age and place of residence.

Lublin is the ninth-largest city in Poland, with over 340,000 inhabitants, and therefore, it can be representative of other Polish larger agglomerations. Our data showed that the significantly highest percentage of virus detection was among people living in Lublin and the lowest among people living in a small town (*p* < 0.0001) (Figure 1). This may be due to the fact that epidemics of diseases spread by airborne droplets proceed faster in densely populated places [9,10]. Moreover, materials were collected in the area of Lublin, which also contributes to a higher number of reported cases among Lublin residents. The reduced number of SARS-CoV-2 infections in smaller towns and villages may be due to poorer access to the health care system and diagnostic centers [11].

**Figure 2:**
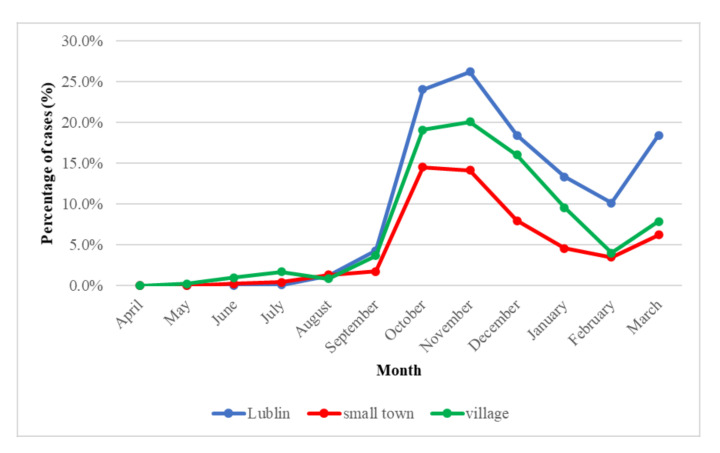
SARS CoV-2 infection from April 2020 to March 2021 in south-eastern Poland by place of residence.

In Poland, during the partial freezing of the economy, the number of extra-family contacts was significantly reduced, and at the end of April 2020, the number of people contacted by a person infected with COVID-19 was almost three people. Along with the loosening of sanitary restrictions, the number of people in “sanitary contact” began to increase rapidly, and in August 2020, this number increased to an average of eight, which created favorable conditions for the development of the epidemic [12]. In our research, we observed a visible increase in infection in September and October in the studied subjects (Figure 2). In addition, a renewed upward trend can be seen in March. Our data shows a similarity in the upward and downward trend of the positive cases with the publicly available data [8]. Salzberger et al. [13] indicated that the number of SARS-CoV-2 infections in most countries is the highest in the 20–59 age group. Our data showed that SARS-CoV-2 was significantly most often detected in the age groups 10–19, 20–29, 30–39, and 90+ (*p* = 0.0001).

**Figure 3:**
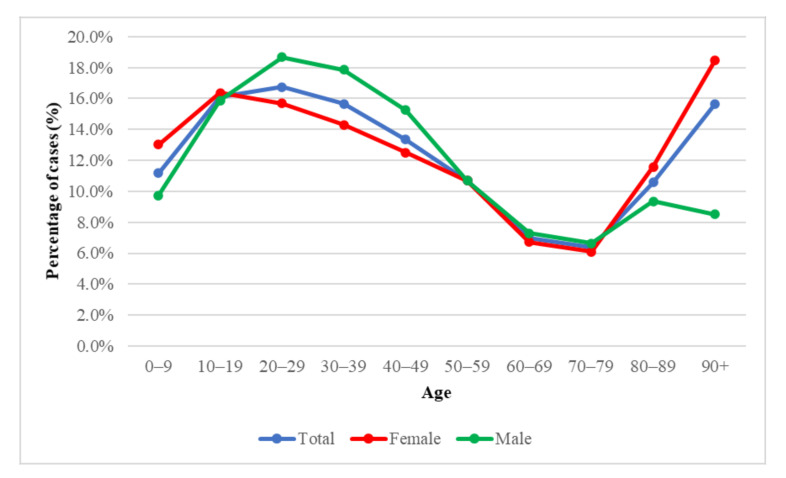
SARS CoV-2 infection by age and sex.

Global Health 50/50 and WHO data reported a similar number of cases of SARS-CoV-2 infection in women and men [3,14]. In addition, there have been more deaths in males in most countries, including those with increased infections in females [14,15]. In the present study (Figure 3) as in the publicly available data, no significant relationship between gender and the frequency of virus detection was observed.

**Figure 4:**
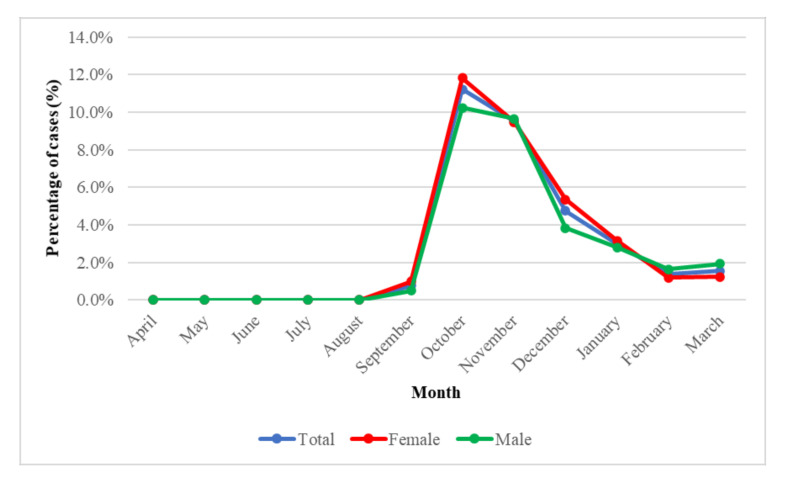
SARS CoV-2 infection in oncology patients (April 2020–March 2021).

**Figure 5:**
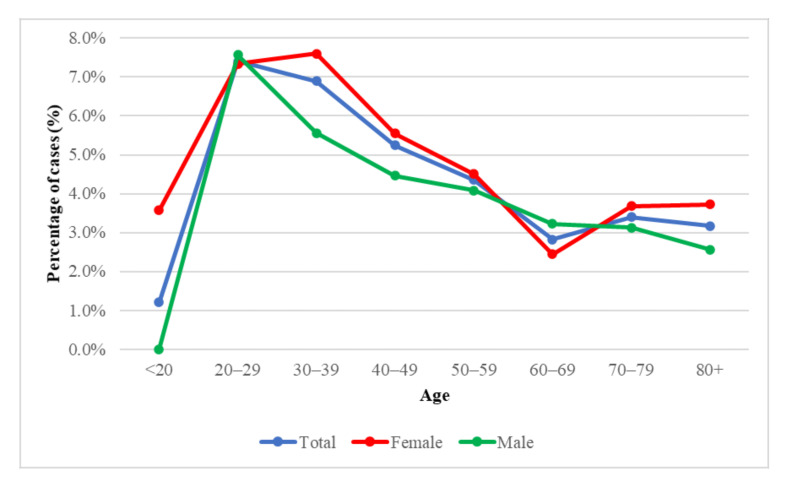
SARS CoV-2 infection in oncology patients by age and sex.

Our study presents the number of COVID-19 infections in cancer patients between April 2020 and March 2021 (Figure 4). In addition, data on cases of COVID-19 infection in cancer patients by gender and age were provided (Figure 5). Many studies have shown that older age and comorbidities, such as cancer, are risk factors for severe disease, complications, and death from COVID-19 [16,17]. For example, Gujski et al. [5] showed that older age, male gender. and the presence of comorbidities is associated with the risk of in-hospital death associated with COVID-19. Liang et al. [18] found that patients infected with SARS-CoV-2 have a higher risk and incidence of serious events compared to non-cancer patients. Moreover, the China Centers for Disease Control and Prevention noted that 5.6% of the death rate of COVID-19 patients was related to cancer patients [19,20]. Our study showed that the number of infections in cancer patients was significantly lower (*p* = 0.0001) than in non-cancer patients. Cancer patients are among the most susceptible to COVID-19 infection, as reported in the media. Therefore, the fear of visiting medical facilities and public places could have contributed to lower infection rates. The reduced number of infections in cancer patients may also be the result of taking special precautions by these patients, for example, by wearing masks and strict adherence to the hygiene of the hands and respiratory tract. In the case of cancer patients as well as non-oncology patients, the highest number of positive cases was observed in October and November, and the first increase in infections was reported in September [21].

Health authorities, such as WHO or the Centers for Disease Control and Prevention (CDC), have recommended mass and rapid tests for the diagnosis of SARS-CoV-2 infection [3,22,23]. The real-time PCR method is widely used in the diagnosis of SARS-CoV-2 due to its high sensitivity, specificity, and the ability to test numerous samples at the same time [11,24]. Our study used an automated RNA-isolation method that reduces errors caused by the “human factor”. It should be noted that the RT-PCR method has several limitations, including the possibility of obtaining a false-negative result, which in the case of patients with high clinical suspicion, requires many tests to confirm the results [25]. In addition, the number of tests in individual countries in the world varies, which has an impact on the number of positive cases [11,26].

Limitations of these studies also include the lack of information on fatalities and other comorbidities in the patients studied. On the other hand, our study was conducted on numerous people, including cancer patients, all age groups, and symptomatic or asymptomatic patients. In the face of the forecast of the next fourth wave of the pandemic in Poland, our study may provide important epidemiological information on the number of positive COVID-19 cases.

## 5. Conclusions

Our study showed that the significantly highest percentage of virus detection was among people living in Lublin and the lowest among people living in a small town (*p* < 0.0001). There was no significant relationship between gender and the virus-detection rate. The virus was significantly most often detected in the age groups 10–19, 20–29, 30–39, and 90+ (*p* = 0.0001). Among oncological patients, the percentage of positive results was significantly lower than in non-oncological patients (*p* = 0.0001).

## Figures and Tables

**Table 1:** Socio-demographical structure of tested population.

Variables		*n* (%)
**Total**		44,801 (100.0%)
**Sex**	**Male**	18,337 (40.93%)
	**Female**	26,453 (59.05%)
	**Not available**	11 (0.02%)
**Age**	**0–9**	1162 (2.59%)
	**10–19**	1085 (2.42%)
	**20–29**	4010 (8.95%)
	**30–39**	5175 (11.55%)
	**40–49**	6756 (15.08%)
	**50–59**	8090 (18.06%)
	**60–69**	9862 (22.01%)
	**70–79**	5967 (13.32%)
	**80–89**	1692 (3.78%)
	**90+**	166 (0.37%)
	**Not available**	836 (1.87%)
**Place of residence**	**Lublin**	20,493 (45.74%)
	**Small town**	9149 (20.42%)
	**Village**	14,929 (33.32%)
	**Not available**	230 (0.51%)
**Patients**	**Oncological**	14,970 (33.41%)
	**Non-oncological**	29,831 (66.59%)

**Table 2:** Detection of SARS Cov-2 depending on socio-demographical variable.

	SARS Cov-2 Positive	SARS Cov-2 Negative	Pearson’s Chi-Square Test
**Sex**			Chi-square = 0.898; *p* = 0.343
**Male**	2045 (11.15%)	16,292 (88.85%)	
**Female**	2873 (10.86%)	23,580 (89.14%)	
**Age**			Chi-square = 614.898; *p* = 0.000
**0–9**	130 (11.19%)	1032 (88.81%)	
**10–19**	175 (16.13%)	910 (83.87%)	
**20–29**	671 (16.73%)	3339 (83.27%)	
**30–39**	811 (15.67%)	4364 (84.33%)	
**40–49**	901 (13.34%)	5855 (86.66%)	
**50–59**	864 (10.68%)	7226 (89.32%)	
**60–69**	690 (7.0%)	9172 (93.0%)	
**70–79**	380 (6.37%)	5587 (93.63%)	
**80–89**	179 (10.58%)	1513 (89.42%)	
**90+**	26 (15.66%)	140 (84.34%)	
**Place of residence**			Chi-square = 483.084; *p* = 0.000
**Lublin**	2902 (14.16%)	17,591 (85.84%)	
**Small town**	523 (5.72%)	8626 (94.28%)	
**Village**	1463 (9.80%)	13,466 (90.20%)	
**Patients**			Chi-square = 1033.801; *p* = 0.000
**Oncological**	627 (4.19%)	14,343 (95.81%)	
**Non-oncological**	4239 (14.21%)	25,592 (85.79%)	
